# Supplementary material for: Urban forests sustain diverse carrion beetle assemblages in the New York City metropolitan area
Source: PeerJ. 2017 Mar 15;5:e3088. doi: 10.7717/peerj.3088 (PMC5356479; doi:10.7717/peerj.3088)
Supplement: Table S1 — Equations retrieved from (Jost, 2006). [file peerj-05-3088-s001.docx]

**Table S1.** Hill’s True Diversity numbers; q=0, q=1 and q=2 calculated for comparison of species diversity indices. Equations retrieved from Jost (2006).

| **Site** |  | **Species Richness**  **(q=0)** |  | **Shannon Entropy**  **(q=1)** | **Simpson’s Reciprocal Index**  **(q=2)** |
| --- | --- | --- | --- | --- | --- |
|  |  | $\sum_{i=1}^{s} p_{i}^{0}$ |  | $exp(-\sum_{i=1}^{s} p_{i}\ln p_{i})$ | $\frac{1}{\sum_{i=1}^{s} p_{i}^{2}}$ |
| NYBG |  | 6 |  | 3.592 | 2.924 |
| HBP |  | 6 |  | 2.310 | 1.804 |
| CP |  | 2 |  | 1.735 | 1.574 |
| IHP |  | 6 |  | 2.970 | 2.628 |
| VCP |  | 5 |  | 2.864 | 2.313 |
| SWP |  | 5 |  | 2.738 | 2.353 |
| LCC |  | 5 |  | 2.328 | 1.742 |
| CSH |  | 5 |  | 3.415 | 2.796 |
| RSP |  | 4 |  | 2.895 | 2.303 |
| MRG |  | 3 |  | 2.548 | 2.363 |
| CT |  | 5 |  | 2.268 | 1.679 |
| CAT |  | 4 |  | 2.221 | 1.763 |
| CFP |  | 6 |  | 3.208 | 2.569 |
