# Supplementary material for: Urban forests sustain diverse carrion beetle assemblages in the New York City metropolitan area
Source: PeerJ. 2017 Mar 15;5:e3088. doi: 10.7717/peerj.3088 (PMC5356479; doi:10.7717/peerj.3088)
Supplement: Table S2 [file peerj-05-3088-s002.docx]

**Table S2**. Relative abundance (mean) across different site classification (urban, suburban, rural) comparing beetle size classes (small, medium, large) and habitat specialization (generalist, specialist; classifications from Gibbs & Stanton, 2001).

| **Characteristic** | **Relative Abundance (mean)** | | | **ANOVA** | |
| --- | --- | --- | --- | --- | --- |
|  | **Urban** | **Suburban** | **Rural** | **F** | **p** |
| ***Beetle Size*** |  |  |  |  |  |
| **Small (<5mm)** | 0.253 | 0.273 | 0.356 | 0.216 | 0.807 |
| **Medium (6-6.5mm)** | 0.121 | 0.089 | 0.049 | 2.948 | 0.057 |
| **Large (>6.5mm)** | 0.253 | 0.273 | 0.356 | 2.502 | 0.104 |
|  |  |  |  |  |  |
| ***Habitat Specialization*** |  |  |  |  |  |
| **Generalist** | 0.256 | 0.282 | 0.340 | 0.138 | 0.872 |
| **Specialist** | 0.081 | 0.073 | 0.053 | 0.244 | 0.784 |
|  |  |  |  |  |  |
