# Supplementary material for: Urban forests sustain diverse carrion beetle assemblages in the New York City metropolitan area
Source: PeerJ. 2017 Mar 15;5:e3088. doi: 10.7717/peerj.3088 (PMC5356479; doi:10.7717/peerj.3088)

**Figure S1.** Relationship between carrion beetle (A) species richness and (B) species diversity (1/D) with percent forest area at all sampled sites (site abbreviations found in Table 1, Figure 1).

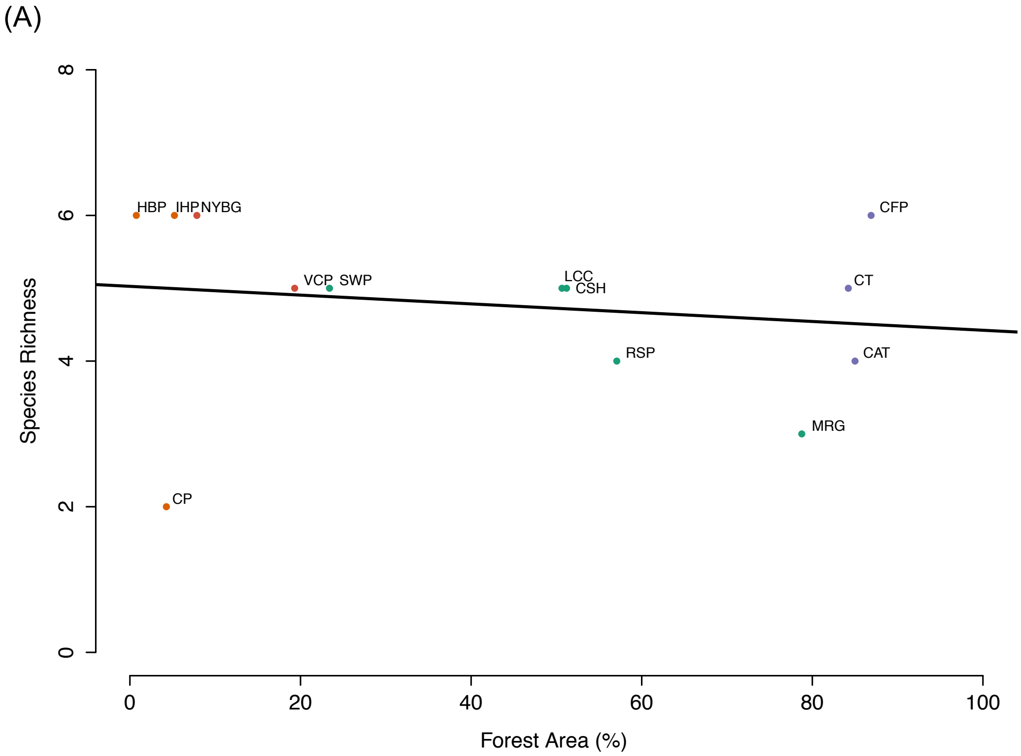


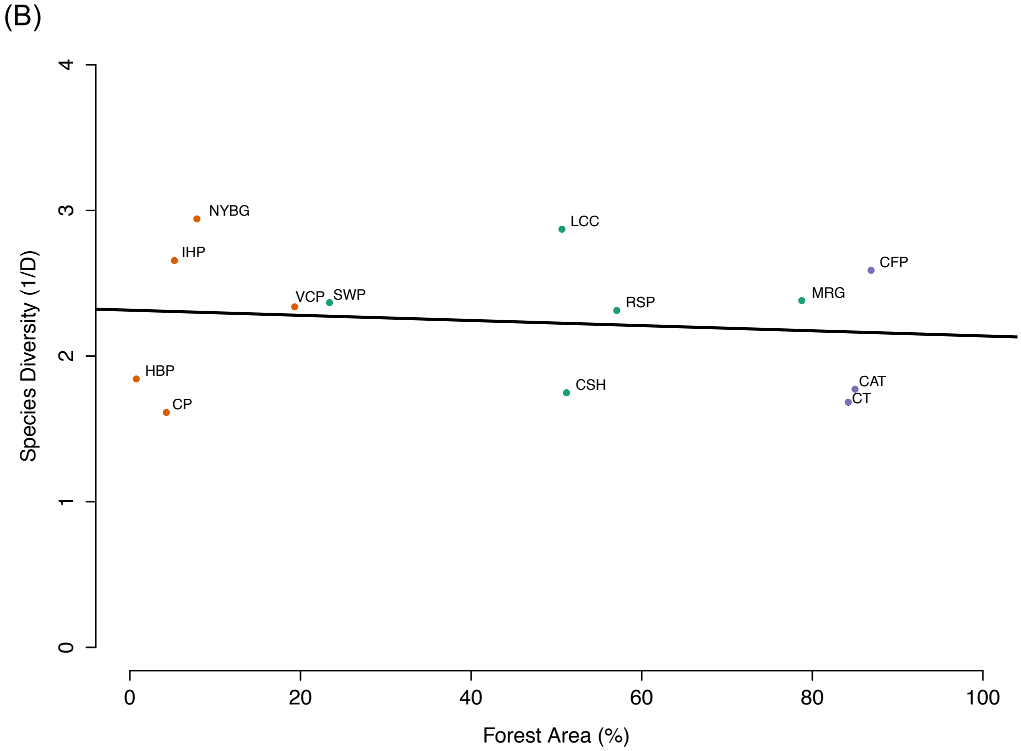

Supplement: Figure S1 [file peerj-05-3088-s003.pdf]
